# Data set on professional attraction and entrepreneurial intention of students in a selected Nigerian University

**DOI:** 10.1016/j.dib.2018.06.009

**Published:** 2018-06-14

**Authors:** A. Omotayo Osibanjo, Maxwell Olokundun, Ovidiu Bordean, Anthonia Adeniji, Salau Odunayo, Stephen Ibidunni

**Affiliations:** a Covenant University, Nigeria; b Babeş-Bolyai University, Romania

**Keywords:** Professional attraction, Entrepreneurial intention, University students Nigeria

## Abstract

This article presents data on the effect of professional attraction to entrepreneurship and the development of entrepreneurial intentions by university students using Covenant University in Nigeria as the case study. The study employed a descriptive quantitative research design by means of survey method. The population of the study comprised all students in the selected university with a total of 7988 students. A sample size of 400 students was selected. Reliability and validity measures were established. Data was analyzed employing Statistical Package for Social Sciences (SPSS). Regression analysis was used as statistical tool of analysis. The analyzed field data set is presented in this article.

**Specification Table**

**Table:** 

**Subject area**	Business, Management
**More Specific Subject Area**	Human Resource Management, Entrepreneurship
**Type of Data**	Table
**How Data was Acquired**	Customer researcher questionnaire
**Data format**	Raw, analyzed, Inferential statistical data
**Experimental Factors**	Sample consisted of university students. The researcher-made questionnaire which contained data on professional attraction and entrepreneurial intention of university were completed.
**Experimental features**	Professional attraction is an important component of entrepreneurial development of students in universities
**Data source location**	South west Nigeria
**Data Accessibility**	Data is included in this article

**Value of data**

● These data present information on professional attraction as it relates to entrepreneurial intentions of students in the university context. This is important considering that professional attraction to a career in entrepreneurship reinforces the propensity for development of entrepreneurial intentions.
● The data set showed that fostering professional attraction for entrepreneurship in universities has implications for job creation potentials by university students.
● The data set can motivate the identification of relevant professional attraction indices required to stimulate the development of entrepreneurial intentions by university students.

## 1 Data

The data comprised inferential statistical data on professional attraction and entrepreneurial intentions of students of covenant university Nigeria. Professional attraction in this context refers to the extent to which students hold a positive or negative assessment of engaging in entrepreneurship. Entrepreneurial intention also refers to the efforts of a student in carrying out entrepreneurial behavior. Specifically, regression analysis was employed to test the hypothesis proposed. Table 1 shows the model summary of the analysis based on the hypothesis tested. The analysis revealed that professional attraction explained 34.6% variance in entrepreneurial intention (*R*^2^=0.346, *p*<0.05). Showing that the more university students are attracted to entrepreneurship as a career the greater the propensity to engage in efforts towards carrying out entrepreneurial behavior while in school.

**Table 1:** Model summary.

Model	*R*	*R* Square	Adjusted *R* Square	Standard error of the estimate
1	0.589^a^	0.346	0.343	0.97729

a Predictors: (Constant), Professional attraction.

**H**_**01:**_ Professional attraction does not affect entrepreneurial intention.

Table 2 shows Analysis of Variance. Table showing the statistical significance of the results gotten. The Analysis of Variance table relates the variance of the residuals from the regression model to the variance of the original data. The model showed the effect of professional attraction on entrepreneurial intention. The *F*-value is calculated as the Mean Square Regression (98.196) divided by the Mean Square Residual (0.955), yielding *F*=102.812. From this result, the model in the table is statistically significant (Sig=0.000).

**Table 2:** Analysis of variance^a^.

Model		Sum of squares	Degree of freedom	Mean square	*F*	Significance
1	Regression	98.196	1	98.196	102.812	0.000^b^
	Residual	185.289	194	0.955		
	Total	283.485	195			

a Dependent variable: Entrepreneurial intention.

b Predictors: (Constant), Professional attraction.

Table 3 shows regression coefficients. The regression coefficient offers information on the predictable variation in the dependent variable based on a one-unit increase in the independent variable. Based on the results the table below revealed the contributions of professional attraction on entrepreneurial intention and the levels of significance. (Professional attraction; *β*=0.589; *t*=10.140; *p*<0.05).

**Table 3:** Coefficients^a^.

Model		Unstandardized Coefficients		Standardized Coefficients	*T*	Significance	95.0% confidence interval for B	
		B	Standard Error	Beta			Lower Bound	Upper Bound
1	(Constant)	1.217	0.331		3.672	.000	0.563	1.870
	Professional attraction	0.596	0.059	0.589	10.140	.000	0.480	0.712

a Dependent variable: Entrepreneurial intention.

## 2 Experimental design, materials and methods

The data presented a quantitative research based on a descriptive research design to assess the effect of professional attraction on entrepreneurial intentions of university students [1], [2], [6]. Survey method was deemed suitable for data collection. Covenant University was selected from South west Nigeria [3]. The study population included all students of Covenant University, which has a population of 7898 students. A randomly chosen set of 400 students participated in this research. The sample size was derived using the Ref. [1] formula. According to Ref. [1], the sample size for a research study can be determined through the following presentation:

From the estimation, a population of 7898 students at alpha value of 0.05 will produce a sample size of 367 respondents. Hence, 400 respondents were used to make room for lost copies of questionnaire or the ones that are not well completed. Data were collected from students across the various colleges in the selected university using a customized researcher- made questionnaire [4], [5]. The data was coded and inputted in SPSS version 25. Data was analyzed employing inferential statistical tests involving regression analysis (Table 4).

**Table 4:** Determination of sample size.

Populationsize	Sample size					
	Continuous data			Categorical data		
	(margin of error=0.03)			(margin of error=0.05)		
	alpha=0.10	alpha=0.05	alpha=0.01	*p*=0.50	*p*=0.50	*p*=0.50
	*t*=1.65	*t*=1.96	*t*=2.58	*t*=1.65	*t*=1.96	*t*=2.58
100	46	55	68	74	80	87
200	59	75	102	116	132	154
300	65	85	123	143	169	207
400	69	92	137	162	196	250
500	72	96	147	176	218	286
600	73	100	155	187	235	316
700	75	102	161	196	249	341
800	76	104	166	203	260	363
900	76	105	170	209	270	382
1000	77	106	173	213	278	399
1500	79	110	183	230	306	461
2000	83	112	189	239	323	499
4000	83	119	198	254	351	570
6000	83	119	209	259	362	598
8000	83	119	209	262	367	613
10,000	83	119	209	264	370	623
